# An Integral-Direct GOSTSHYP Algorithm for the Computation of High Pressure Effects on Molecular and Electronic Structure

**DOI:** 10.1021/acs.jctc.4c01502

**Published:** 2025-01-17

**Authors:** Ansgar Pausch, Felix Zeller, Tim Neudecker

**Affiliations:** † Theoretical Chemistry, Vrije Universiteit, 1081HV Amsterdam, The Netherlands; ‡ University of Bremen, Institute for Physical and Theoretical Chemistry, Leobener Str. 6, D-28359 Bremen, Germany; § Bremen Center for Computational Materials Science, University of Bremen, Am Fallturm 1, D-28359 Bremen, Germany; ∥ MAPEX Center for Materials and Processes, University of Bremen, Bibliothekstr. 1, D-28359 Bremen, Germany

## Abstract

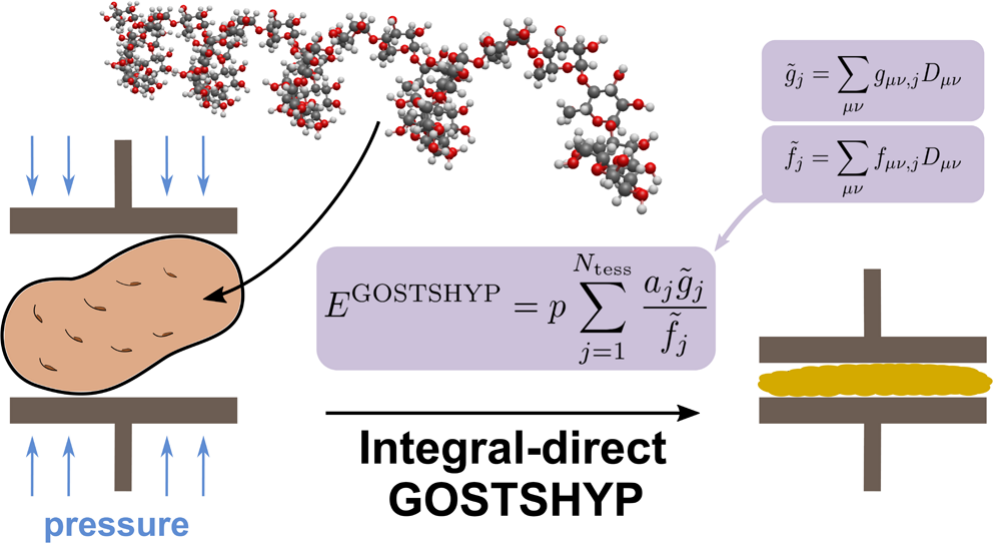

To simulate the effects of high pressure on molecular and electronic structure, methods based on the polarizable continuum model have emerged as a serious contender to the conventionally employed periodic boundary conditions. In this work, we present a highly efficient integral-direct algorithm for the Gaussians On Surface Tesserae Simulate HYdrostatic Pressure (GOSTSHYP) method. We examine the efficiency of this implementation on large chains of α-d-glucose units. Furthermore, we investigate the effects of high pressure on the binding energy of a supersystem consisting of a buckminster fullerene and a corannulene pincer system, and juxtapose various types of surfaces that constitute the boundary between the molecule and the implicit solvent. Our efficient implementation of the GOSTSHYP model paves the way for large-scale simulations of molecules under pressure.

## 1 Introduction

Pressure in the gigapascal (GPa) range leads to many astonishing phenomena, such as the appearance of new crystal structures,^1−3^ changes of spectroscopic properties,^4−6^ changes in reaction thermodynamics and kinetics,^7,8^ decompositions,^9^ or amorphizations.^9,10^ The development of the diamond anvil cell^6,11,12^ nowadays allows for the experimental investigation of such phenomena for pressures of up to several hundred GPa.^13^

Computationally, high pressure effects, such as phase transitions and structural changes of periodic systems, are widely studied by the use of plane-wave density functional theory (DFT) with the stress theorem.^14−23^ Here, increasing pressure leads to a smaller unit cell and thus stronger repulsive interactions between molecules and their environment. To model spectroscopic properties and to analyze bonding situations and electronic structure at high pressure, single molecule methods are often preferable, since they offer a wide pallet of established tools. However, *ab initio* calculations on finite systems under high pressure have to account for the interaction with the surrounding in an approximate manner.

The first *ab initio* studies on single atoms and linear molecules under high pressure conditions used infinite spherical confinement potentials to model the surroundings.^24−28^ While related approaches are still used today in many insightful studies,^29−32^ they are limited to the smallest molecules such as methane. Mechanochemical models such as the generalized force-modified potential energy surface (G-FMPES)^33^ or extended hydrostatic compression force field (X-HCFF)^34^ offer a computationally cheap alternative by adding external compression forces directly to the nuclear gradient during a geometry optimization. However, they neglect the effect of the compression on the electronic structure. In 2008, Cammi et al. presented the extreme-pressure polarizable continuum model (XP-PCM),^35^ which adds a Pauli-repulsion term to the PCM Hamiltonian and shrinks the PCM cavity to model compression. Until today, XP-PCM has seen continuous development^36−40^ and has been successfully used to model pressure effects on, e.g., molecular vibrations^41,42^ and organic reactions.^43,44^ For a more detailed discussion of the aforementioned models we refer the interested reader to our recent review.^45^

Recently, an alternative approach was proposed: the Gaussians On Surface Tesserae Simulate HYdrostatic Pressure (GOSTSHYP) model, in which a molecule is surrounded by repulsive Gaussian potentials located on a discretized cavity.^46,47^ The potentials scale with pressure and add a compressing term to the molecular Hamiltonian. This approach is very promising, as it allows for the inclusion of pressure effects into any quantum chemical method without having to use periodic boundary conditions. As such, GOSTSHYP can be used to investigate the effects of high pressure on not just molecular structure, but rather any electronic or spectroscopic property. Furthermore, the striking similarities between GOSTSHYP and the widely established COnductor-like Screening MOdel (COSMO)^48−50^ suggest that these methods can use the same infrastructure within quantum chemical software. Therefore, the implementation of GOSTSHYP into established software is generally straightforward.

However, two major problems have plagued previous GOSTSHYP implementations.^46,47^ First, the calculation of increasingly large molecular systems was hindered by exceedingly large memory requirements due to the unfavorable  scaling of intermediate quantities, with *N*_basis_ being the number of basis functions and *N*_tess_ the number of tesserae which are used to discretize the cavity. In order to tackle this issue, a sophisticated integral screening algorithm was presented.^47^ While this allows for the calculation of larger systems, the overall scaling of intermediate quantities remains unfavorable, particularly with respect to an increasing basis set size. Second, van der Waals (vdW) surfaces were exclusively used to describe the GOSTSHYP cavity. In most cases, vdW surfaces discretized using Lebedev grids are a convenient choice to model the cavity as they allow for fully consistent derivatives.^51−53^ However, vdW surfaces are also associated with the emergence of undesirable crevices that are known to lead to significant problems for the COSMO in some cases.^54^ For GOSTSHYP, these crevices can lead to ill-defined amplitudes for the model Gaussian potentials, which result in severe convergence problems for self-consistent field (SCF) methods.^47^

In this work, we present an efficient implementation of GOSTSHYP that is not plagued by these problems. Using the existent infrastructure for COSMO, we have implemented an integral-direct version of GOSTSHYP into the Turbomole(55−57) program suite. The integral-direct algorithm reduces the memory requirement from  to , making the memory demand no longer a computational bottleneck. Furthermore, all cavities previously available with the Turbomole program suite can now also be used for GOSTSHYP. This includes those which use a solvent-excluded surface or (pseudo)isodensity surfaces, which are less likely to include the undesirable crevices that can lead to the aforementioned convergence issues. The importance of choosing a suitable cavity for GOSTSHYP was very recently highlighted by Eeckhoudt, Alonso, Geerlings, and De Proft in ref (58).

This work is structured as follows. In Section 2, we describe the theoretical intricacies of an integral-direct GOSTSHYP algorithm and briefly discuss its implementation for SCF modules as well as for geometry gradients. We conclude the section by examining the different choices of cavity which are now available for GOSTSHYP. Section 3 gives a brief overview of computational methods. In Section 4 we demonstrate the efficiency of our implementation by examining extended molecular systems. Here, we perform benchmark calculations on increasingly large chains of α-d-glucose units. Finally, we investigate the effects of the cavity choice on a supersystem consisting of the C_60_ fullerene and a corannulene pincer system. In particular, we assess the cavity effects on the binding energy. We conclude the work in Section 5. Appendix A contains some more details on the integral evaluation and an efficient approximate screening technique for GOSTSHYP.

## 2 Theory and Implementation

### 2.1 Model Potentials

In GOSTSHYP, the effects of hydrostatic pressure on a molecular system are introduced self-consistently into quantum chemical calculations.^46^ To achieve this, a suitable cavity is chosen and parametrized through a set of surface segments typically referred to as tesserae. Gaussian potentials of the form
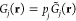
1are placed on the centers of each tessera *j*, with *p*_*j*_ being their respective amplitudes and
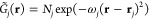
2being normalized Gaussian potentials. Only the amplitudes are self-consistently optimized, all other parameters remain constant during an SCF procedure. This includes centers of the tessera **r**_*j*_, exponents ω_*j*_, as well as normalization constants:
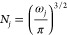
3In order to get relatively homogeneous potentials surrounding the molecule, we require that the potential is halved at the edge of each (circular) tessera
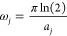
4where *a*_*j*_ is the area of a tessera. It should be highlighted that the positions and areas of tesserae are the only cavity parameters that enter the GOSTSHYP equations. Furthermore, it should be noted that the normalization in eq 3 is not strictly necessary and was not used in previous works.^46,47^ However, in Section A.2 of this work, we will demonstrate that the use of normalized Gaussians leads to a more stable algorithm that allows for a straightforward screening of GOSTSHYP integrals for extended molecular structures.

### 2.2 GOSTSHYP Energy

A detailed derivation of the working equations in GOSTSHYP has been presented in ref (46). Here, we will focus on how to construct all relevant quantities using an integral-direct algorithm. The GOSTSHYP energy contribution is constructed as a sum over all individual contributions^46,47^
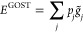
5The similarity to the construction of the COSMO energy is striking.^48^ Instead of COSMO charges, we need to self-consistently construct a vector of GOSTSHYP amplitudes *p*_*j*_ and instead of a COSMO potential, an auxiliary vector of normalized GOSTSHYP potentials *g̃*_*j*_ is assembled. The semiclassical pressure equations lead to the following expression for the amplitudes^46^
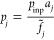
6In addition to the user-defined input pressure *p*_inp_, the amplitudes only depend on the tessera’s area *a*_*j*_ and elements of an auxiliary vector *f̃*_*j*_. Both auxiliary vectors **g̃** and **f̃** are evaluated through a contraction of three-center integrals with the density matrix
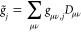
7
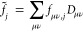
8For an integral-direct algorithm, these two auxiliary vectors need to be evaluated in every iteration. In practice, the three-center integrals in (eqs 7 and 8) are calculated in batches and contracted with batches of the density matrix on the fly. For basis sets consisting of Gaussian-type orbitals (GTOs), the integral *g*_μν,*j*_ can be evaluated as a three-center overlap of Gaussian functions using the Gaussian product theorem. Here, we use the short-hand notation
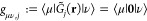
9The GOSTSHYP Gaussian function can be interpreted as a normalized *s*-type Cartesian GTO centered at **r**_*j*_. The associated angular momentum quantum number is |**l**_*G̃*_| = 0, leading to the above notation. A variety of different algorithms can be used to evaluate such a type of integral. In this work, we have implemented a Gauss–Hermite polynomial approach.

The *f*_μν,*j*_ integrals are constructed as a scalar product of a segment’s normal vector **n**_*j*_ and the gradient of the *g*_μν,*j*_ integral
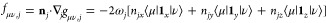
10As such, three-center overlap integrals with *p*-type GOSTSHYP Gaussians need to be evaluated. Both the *g*_μν,*j*_ and *f*_μν,*j*_ integrals can be computed in batches simultaneously due to their similar structures, reducing the computational effort.

We close this section by remarking that the GOSTSHYP energy contribution in eq 5 is very similar to the *pV* (*pressure* times *volume*) term used in standard definitions of the enthalpy. The effective volume *V*^eff^ associated with the GOSTSHYP method could be interpreted as
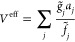
11Please note that this effective volume is not equivalent to the volume of the cavity. It is generally not advised to interpret the total electronic energy within the GOSTSHYP model as the system’s enthalpy, despite their similarity. In GOSTSHYP, pressure effects on an individual molecular structure can be evaluated, while thermodynamical quantities such as the enthalpy refer to an ensemble of particles.

### 2.3 Fock Matrix Contribution

In order to self-consistently calculate the GOSTSHYP energy, the Fock matrix contribution has to be evaluated in every iteration of the SCF procedure. It is constructed as the derivative of the GOSTSHYP energy with respect to the density matrix, which yields
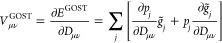
12A more convenient form of the first term can be obtained via the chain rule of derivatives
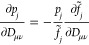
13whereas the respective derivatives of the auxiliary vectors are obtained as
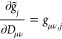
14
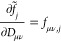
15Assembling all of this, we can write out the GOSTSHYP Fock matrix contribution^46^
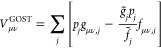
16

### 2.4 Implementation of the SCF Algorithm

We have implemented the integral-direct GOSTSHYP algorithm for SCF calculations into the Turbomole(55−57) program suite, where it will be available in the next official release version (V7.9). The correctness of our implementation was confirmed by comparison to an existing GOSTSHYP implementation in Q-Chem.^59^ Both implementations agree up to machine precision with one another. In the remainder of this section, we briefly summarize a few more details on our integral-direct SCF algorithm. As previously mentioned, the auxiliary vectors **g̃** and **f̃** need to be evaluated in every iteration through a contraction with the density matrix. Even though an efficient integral screening and a subsequent contraction in batches can significantly reduce the computational effort, this is generally the most time-consuming GOSTSHYP step in the SCF procedure. We will demonstrate this in Section 4.1. The amplitude vector and the electronic energy can be obtained from the auxiliary vectors without further computational effort.

**Figure 1:**
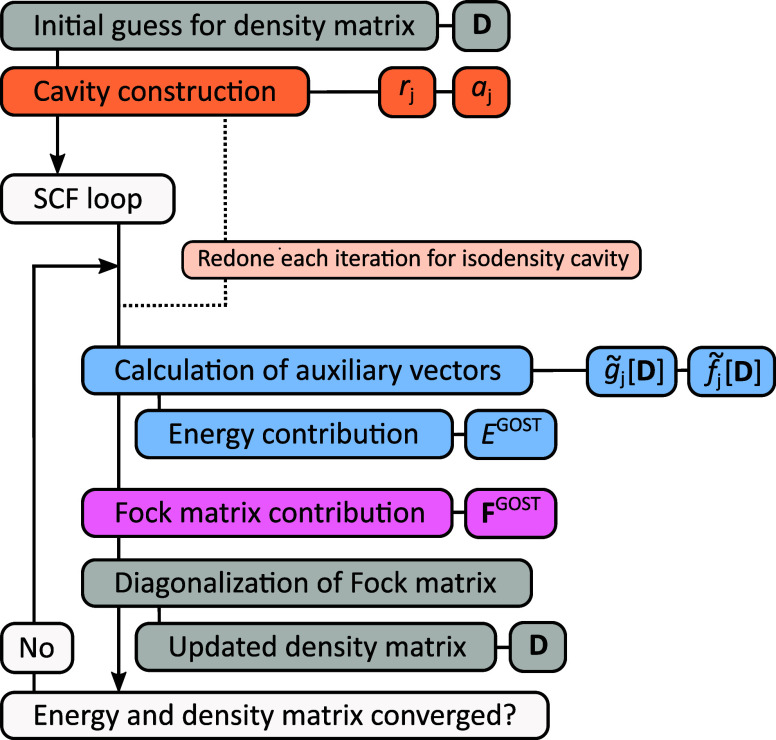
Schematic overview of the relevant GOSTSHYP steps in the SCF procedure. This includes the cavity construction (orange), calculation of auxiliary vectors (blue) and the Fock matrix contribution (pink). All three-center integrals *g*_μν,*j*_ and *f*_μν,*j*_ have to be calculated twice per iteration (in the blue and pink sections, respectively). Different types of cavities can be used (see Section 2.6), but most are constructed only once, at the beginning of the SCF procedure. The isodensity cavity is an exception, as it has to be newly constructed in each iteration.

In a subsequent step, the Fock matrix contribution of eq 16 is constructed. This requires us to keep the auxiliary vectors **g̃** and **f̃** in memory and to then calculate all three-center integrals *g*_μν,*j*_ and *f*_μν,*j*_ an additional time, evaluating the sum over all tesserae in eq 16 as the innermost step. Batches of the GOSTSHYP contribution to the Fock matrix calculated in this manner are then added to the Fock matrix on the fly, thereby minimizing the memory requirement. A schematic workflow of this method is depicted in Figure 1.

To reiterate, an integral-direct algorithm requires us to compute all GOSTSHYP integrals twice in each iteration of the SCF procedure—once for the construction of the auxiliary vectors and once for the evaluation of the Fock matrix contribution. However, this approach solves the memory issues otherwise associated with the method,^47^ making the calculation of in principle arbitrarily large systems possible. This procedure is equivalent to the integral-direct COSMO implementations established in the literature.^60^

### 2.5 Analytical Geometry Gradients

Analytical GOSTSHYP geometry gradients can be constructed after the SCF procedure has converged. As detailed in Section 2.1, GOSTSHYP only introduces the positions and areas of tesserae as additional parameters into the SCF procedure. As such, we form the full analytical gradient using the chain rule of derivatives with respect to these quantities. Here, the modular structure of the integral-direct algorithm is beneficial, as most quantities can be precomputed and stored in memory. Analytical GOSTSHYP geometry gradients are available with both Hartree–Fock and DFT in our implementation.

The partial derivative of the GOSTSHYP energy with respect to a nuclear displacement can be written as
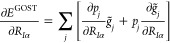
17Here, *R*_*I*α_ refers to the coordinate of nucleus *I* in the α ∈ {*x*,*y*,*z*} Cartesian component. The derivative of the amplitudes may be constructed using the chain rule of derivatives
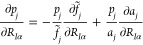
18which includes the cavity derivatives in the form of an area-dependency as well as derivatives of the auxiliary vectors.

Let us focus on the auxiliary vectors first. Derivatives of the density matrix ought not be included here as they are generally contained in the energy-weighted density matrix contribution to the gradient. What remains are derivatives of the three-center integrals which can be contracted on the fly with density matrix elements in batches
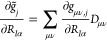
19
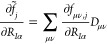
20These can again be separated into two distinct contributions due to the dependency of the exponents ω_*j*_ on the area of the tessera, and thus also on the nuclear displacements. We can write this out as derivatives of the three-center integrals at constant exponents ω_*j*_ as well as a derivative of the three-center integrals with respect to the exponents
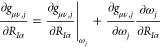
21
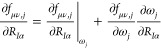
22Here, we can use the chain rule of derivatives to rewrite the latter part as
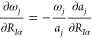
23which can be added onto the previous area-dependent contribution. The derivative of three-center integrals with respect to the exponents leads to new types of integrals, for which we use the short-hand notation
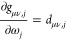
24
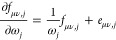
25We neglect the derivatives of the normalization constants *N*_*j*_ in both (eqs 24 and 25) as they cancel out in the full gradient. We are left with two new types of integrals, namely an *s*-type Cartesian *d*-function
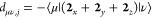
26and a linear combination of *p*-type Cartesian *f*-functions
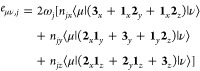
27Since they are directly contracted with the density matrix again in (eqs 19 and 20), we require the following two auxiliary vectors that can be precomputed and stored in memory along their **g̃** and **f̃** counterparts
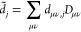
28
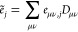
29All of this can be combined in our final expression for the analytical gradient
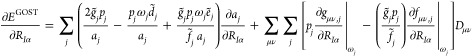
30Please note that no memory-intensive quantities need to be stored for this procedure and all contractions in eq 30 can be performed on the fly. Depending on the chosen cavity, area-derivatives may or may not be available directly—this will be discussed in more detail in Section 2.6.

For the three-center integral derivatives at constant exponents, we can make use of the translational invariance also generally exploited for other three-center integrals such as nuclear attraction or COSMO integrals
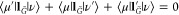
31Here, |**l**_*G̃*_|corresponds to the aforementioned angular momentum quantum number associated with the GOSTSHYP Gaussian. Using this relation, only the derivative of the bra-side needs to be evaluated. This circumvents the rather lengthy steps otherwise resulting from having to form the derivative of the GOSTSHYP Gaussians^46^
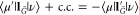
32Further details concerning the integral evaluation are elaborated in Appendix A.1.

### 2.6 Choice of Cavity

In the previous sections, we have discussed all relevant equations for the self-consistent calculation of GOSTSHYP energies and geometry gradients. We shall now return to an important point that was purposefully left ambiguous in the beginning of this discussion: the choice of a suitable cavity. A very recent study^58^ has highlighted the importance of this choice for GOSTSHYP. In this section, we introduce a variety of cavities that have been developed for COSMO within the last few decades. All of these can readily be employed for GOSTSHYP with our current implementation.

In general, finding an appropriate cavity surrounding a molecular system is not a trivial task.^61,62^ First and foremost, the cavity should resemble the system’s environment while not explicitly depending on any outside parameters. This also means that artifacts such as sharp crevices or segments within the molecular structure should not be introduced. The system’s symmetry should be retained when introducing the cavity into calculations. It should be easily constructed, including readily available analytical cavity derivatives. The potential energy surface should remain smooth even if the molecular structure is distorted or dissociated. Unfortunately, no choice of cavity fulfills all of these requirements.

#### 2.6.1 van der Waals Surface

**Figure 2:**
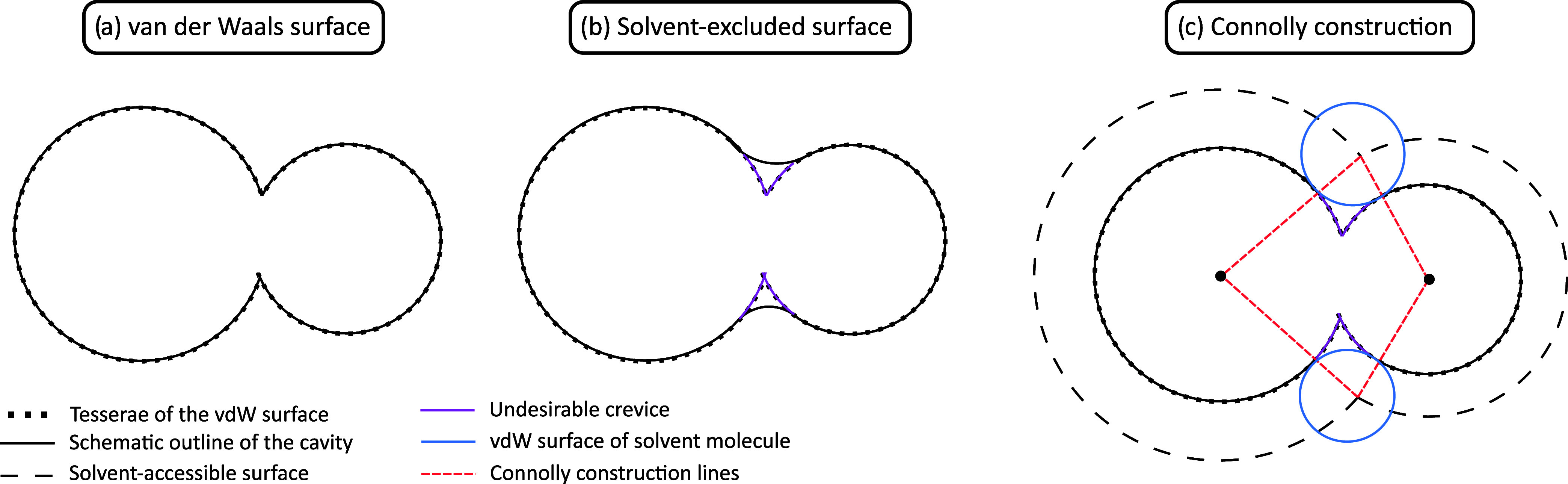
Model depictions of different types of cavities. A van der Waals (vdW) surface is depicted in (a). A solvent-excluded surface (SES) is shown in (b), where the undesirable crevices have been highlighted. The Connolly construction of an SES is shown in (c), including the solvent-accessible surface and the vdW surface of potential solvent molecules. Tesserae discretizing the cavity are indicated via square points.

One common approach is to define van der Waals (vdW) radii *r*_vdW_ for each atom and use the surface enclosed by all of the resulting spheres. This is schematically depicted in (Figure 2a). Such vdW surfaces can be easily constructed and parametrized, for instance through the use of Lebedev grids as suggested by York and Karplus.^51,52,63,64^ For the COSMO, fully consistent analytical second derivatives in this framework have been reported very recently.^53^ However, cavities based on vdW surfaces often include notorious artifacts such as sharp edges, which in some instances can lead to significant problems. For GOSTSHYP, the resulting crevices have been reported to induce ill-defined negative amplitudes *p*_*j*_, thereby leading to very slow convergence of the SCF procedure and potentially even a convergence to different solutions.^46,47^

#### 2.6.2 van der Waals Surface with Outer Cavity Correction

In the context of this work, we have implemented a new approach based on the conventional construction of vdW surfaces. Here, a slightly larger cavity is additionally constructed with an extension radius *r*_ext_. The vdW radius of this outer cavity is defined accordingly as *r*_vdW_^outer^ = *r*_vdW_ + *r*_ext_. Please note that there is a one-to-one mapping of all points on the outer and inner vdW surface if Lebedev grids are used. In the outer cavity correction (OCC) approach, we now use the switching functions of the outer surface for the inner one, thereby screening away the critical points on the undesirable crevices indicated in purple in (Figure 2b). However, we thereby also introduce some small holes in the cavity parametrization. Please note that an increasing number of points are screened away if a larger extension radius is chosen.

#### 2.6.3 Solvent-Excluded Surface

A solvent-excluded surface (SES) refers to the surface of the solute that can be touched by the solvent. This excludes particularly all sharp edges, crevices and other artifacts the vdW surfaces are commonly plagued by. A model of an SES is shown in (Figure 2b). Such an SES can be constructed through a variety of different means.^48,65^ A schematic depiction of the Connolly construction^62^ is presented in (Figure 2c). First, a solvent-accessible surface is constructed and subsequently projected inward to generate the SES. While such a procedure can still lead to ill-defined regions in the resulting cavity in some instances,^54,66^ it generally avoids the more systematic problems of the vdW surface construction. However, due to the intricate nature of the Connolly construction, analytical derivatives of this type of cavity are currently not available in our implementation. As a consequence, geometry gradients are not consistent with the electronic energy.

#### 2.6.4 Isodensity Surface

An entirely different approach presents itself in the construction of an isodensity surface.^67^ In the SCF procedure, the electron density of a molecular structure is updated in every iteration. From this information, an isodensity surface can then be parametrized. It can be self-consistently optimized within the SCF procedure. However, this choice of cavity does not generally retain the system’s symmetry. Furthermore, analytical derivatives are not available, because of the complicated connection between placement of segments and the electronic energy. As a consequence, the resulting PES is typically not smooth as tesserae can (dis-)appear entirely if the molecular structure is distorted.

#### 2.6.5 FINE Cavity

More recently, Diedenhofen and Klamt proposed a cavity based on a pseudoisodensity.^54^ This FINE cavity combines some of the advantages of the isodensity approach with the more straightforward construction of the SES. Thus, analytical derivatives of the segment areas are generally available within this approach. However, a symmetry-adapted construction of the FINE cavity has thus far not been presented in the literature. Similarly to the isodensity surface, the associated PES is typically not smooth, although this appears to be a problem related to the way the basis grid points are combined in order to form tesserae,^54^ not an intrinsic problem of the method.

## 3 Computational Methods

Having discussed the theoretical background of our integral-direct GOSTSHYP implementation, we now assess its efficiency. Our first model system is an *n*-membered chain of α-d-glucose units with *n* = 1, 2, 4, 8, 16, 32, 48, and 64, also referred to as amylose_*n*_.^68^ This is a common model system for examining the scaling of methods, particularly in the framework of density functional theory.^69−72^ All calculations on amylose molecules were performed using the def2-SV(P) basis set.^73^ As density functional approximations, we chose PBE and PBE0.^74^ Medium-sized grids (grid 3) were employed for the numerical integration.^75^ For calculations performed with PBE, the resolution of the identity approximation was used for the Coulomb part (RI-*J*) with a def2-SV(P) auxiliary basis set.^76^ To further accelerate this computational bottleneck, we additionally performed the amylose_*n*_ calculations on the PBE/def2-SV(P) level of theory with the multipole acceleration of the RI-*J* (MARI-*J*).^77^ For GOSTSHYP, we used the FINE cavity with initial solvation radii all set to 2 Å.^54^ For amylose_*n*_ this corresponds to a total number of tesserae of 669 (*n* = 1), 1052 (*n* = 2), 1824 (*n* = 4), 3372 (*n* = 8), 6455 (*n* = 16), 12643 (*n* = 32), 18887 (*n* = 48), and 25008 (*n* = 64), respectively. All self-consistent field calculations were converged up to 10^–7^*E*_h_. Benchmark calculations were run on a single central processing unit (CPU) of the type Intel Xeon E5–2687W v4 @ 3.00 GHz. Furthermore, we also considered the parallel speedup by running calculations on the amylose molecules on more than one CPU.

To determine the impact of different cavities presented in Section 2.6 on GOSTSHYP calculations, we investigated the pressure effect on the binding energy *E*_B_ between the corannulene pincer system and the Buckminster fullerene C_60_. The structures of the isolated and adjoined systems were obtained by optimizing the geometry at the B3LYP/def2-SVP level of theory and employing Grimme’s D3 dispersion correction with Becke-Johnson damping.^73,78−81^ The basis set superposition error (BSSE) was accounted for by employing the counterpoise correction. For all calculations involving the pincer and the adjoined system, medium-sized grids (grid 3) were employed for the numerical integration. However, the high symmetry of C_60_ led to substantial numerical errors using grid 3, consequently a finer grid (grid 5) was used for C_60_.^75^ Self-consistent field calculations were converged up to 10^–7^*E*_h_, geometry optimizations up to an energy change of 10^–6^*E*_h_ and a gradient norm of 10^–3^*E*_h_/Å. These GOSTSHYP calculations were performed at the B3LYP-D3/def2-SVP level of theory.^73,78−81^ The atomic radii for construction of Lebedev grids and the Connolly construction were determined by fitting radii to represent the isodensity surface of the adjoined system, resulting in atomic radii of 1.4 Å for H and 2.1 Å for C. Calculations using the outer cavity correction approach for vdW surfaces used an extension radius of *r*_ext_ = 0.25 Å.

Please note that we have exclusively used basis sets of double-ζ quality throughout this work. For practical calculations of pressure effects on molecules, the flexibility of a larger basis may often be needed to obtain quantitative or even qualitative results.

## 4 Results and Discussion

### 4.1 Efficiency Assessment

**Figure 3:**
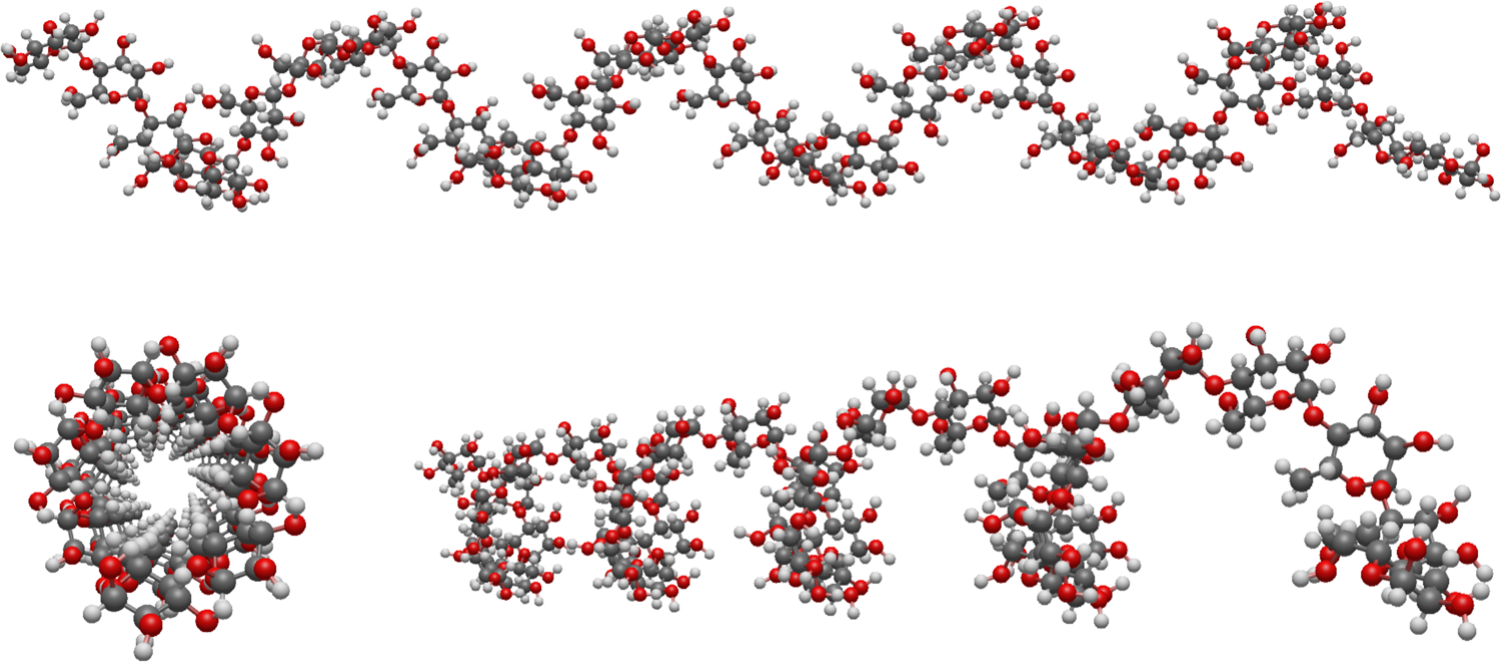
Helix structure of amylose_32_ depicted from different angles. The structure was taken from ref (68).

In order to demonstrate the efficiency of our integral-direct GOSTSHYP implementation, we first analyze the computation times of amylose_*n*_ with up to *n* = 32 using the hybrid functional PBE0. The molecular structure of the model system amylose_32_ is depicted from different angles in Figure 3.^68^ It forms a helix structure with more distant α-d-glucose units being well-separated, making it an ideal system to assess the scaling of a method with respect to system size.

Since PBE0 is a hybrid functional, both the Coulomb and the exact exchange contribution have to be computed. No approximations were used for the two-electron part, other than the commonly employed approximate screening techniques.^82^ For GOSTSHYP, we chose an external pressure of 50 GPa and used the approximate screening technique described in Section A.2 of this work. The timings are presented in Table 1, where both the absolute time and the percentage of that time spent on GOSTSHYP are listed. Furthermore, the GOSTSHYP timings are split up into their respective parts within the integral-direct scheme as indicated by the colors in Figure 1. These three parts are the cavity construction (orange), the first step in which the auxiliary vectors **g̃** and **f̃** are computed (blue), as well as the second step in which the Fock matrix contribution is calculated (pink).

**Table 1:** Computation Time for GOSTSHYP Calculations on Amylose_*n*_, Performed on the PBE0/def2-SV(P) Level of Theory^a^

amylose_*n*_	Time in [s]	GOSTSHYP (%)	cavity (%)	1st step (%)	2nd step (%)
1	80	13.3	1.8	8.2	3.3
2	391	9.3	0.7	6.0	2.6
4	1752	8.8	0.3	5.7	2.8
8	7435	9.2	0.2	5.9	3.1
16	31303	8.8	0.1	5.4	3.2
32	158288	7.6	0.1	4.7	2.8

a Fraction of the overall computation time spent on GOSTSHYP is shown, including the individual fractions of the cavity construction and both GOSTSHYP steps in the integral-direct algorithm.

The first takeaway from the timings listed in Table 1 is that GOSTSHYP never becomes a computational bottleneck in any of the calculations. For all systems except amylose_1_, GOSTSHYP requires less than 10% of the computation time. This percentage generally decreases with an increasing system size, although this effect is not very consistent, with amylose_8_ being a clear outlier. Comparing the timings of the two steps in the integral-direct GOSTSHYP procedure leads to an interesting realization. The first step (construction of the auxiliary vectors **g̃** and **f̃**) is about 2 times more time-consuming than the second step (construction of the Fock matrix contribution). This could come as a surprise as both steps require the calculation of all three-center integrals *g*_μν,*j*_ and *f*_μν,*j*_ in (eqs 9 and 10). Clearly, the calculation of these integrals alone is not the computational bottleneck, but instead their contractions with the density matrix in (eqs 7 and 8) and with the amplitudes in eq 16, respectively. As previously described in Section 2.4, we reduced the computational effort of this step by performing the contraction with the density matrix in shell batches. The approximate screening technique discussed in Section A.2 of this work further reduces the computational effort of this step for large systems, as entire shell batches can be screened out if they have negligible contributions. Overall, the timings in Table 1 demonstrate that for SCF calculations in which exact exchange is calculated, such as Hartree–Fock or (range-separated) hybrid functionals, GOSTSHYP generally does not affect the scaling. Please note that this does not have to be the case anymore if approximations for the exchange part like the resolution of the identity for exchange or seminumerical exchange are employed.^83,84^

Pure density functionals scale much more favorably than their (range-separated) hybrid counterparts, particularly if RI-*J* is used. This reduces the overall scaling of the method from  to .^85^ Here, the scaling of the method becomes similar to the overall scaling of the integral-direct GOSTSHYP algorithm, both of which benefit from approximate integral screening techniques in a similar fashion. As such, we expect GOSTSHYP to take about 50% of the overall computation time for extended molecular systems calculated on the PBE/def2-SV(P) level of theory with RI-*J*. The timings for amylose_*n*_ with up to *n* = 64 are listed in Table 2.

**Table 2:** Computation Time for GOSTSHYP Calculations on Amylose_*n*_, Performed on the PBE/def2-SV(P) Level of Theory, in Combination with RI-*J*^a^

amylose_*n*_	time in [s]	GOSTSHYP (%)	cavity (%)	1st step (%)	2nd step (%)
1	22	45.5	6.5	27.7	11.3
2	82	50.5	3.2	33.1	14.2
4	223	60.9	2.6	39.4	18.9
8	1491	56.5	0.8	36.3	19.4
16	6530	53.5	0.6	33.8	19.2
32	27403	49.4	0.8	30.4	18.3
48	64963	47.7	1.2	28.8	17.8
64	119173	46.7	1.8	27.7	17.2

a Fraction of the overall computation time spent on GOSTSHYP is shown, including the individual fractions of the cavity construction and both GOSTSHYP steps in the integral-direct algorithm.

First and foremost, it should be noted that the total computation times are overall much lower than those previously shown for PBE0/def2-SV(P) in Table 1. As expected, GOSTSHYP makes up a much higher percentage of the CPU time for the lower-scaling method—about 50% overall, with its highest value for amylose_4_ at 60.9%. With an increasing system size, the percentage spent on the GOSTSHYP part decreases significantly, with less than 50% of the computation time required for the amylose_64_ molecule. This indicates that the approximate screening of the three-center overlap integrals becomes slightly more efficient than that of the two-electron integrals for extended molecular structures. Overall, GOSTSHYP introduces at most a factor of about 2 to the already highly efficient DFT calculations that employed density fitting. This means that a system such as amylose_64_ with 1347 atoms in a def2-SV(P) basis with 11154 contracted/19488 primitive basis functions can be routinely calculated using our integral-direct GOSTSHYP algorithm.

In order to get an even more favorable scaling of the DFT calculation, further approximations such as MARI-*J* may be introduced.^77^ The use of multipole-based integral estimates is a well-established tool for the acceleration of quantum chemical calculations for extended molecular structures.^86^ For the amylose_*n*_ systems discussed here, approaches such as MARI-*J* are well-known to reduce the overall scaling.^71^ The GOSTSHYP timings for SCF calculations on the PBE/def2-SV(P) level of theory with MARI-*J* are presented in Table 3.

**Table 3:** Computation Time for GOSTSHYP Calculations on Amylose_*n*_, Performed on the PBE/def2-SV(P) Level of Theory, in Combination with MARI-*J*^a^

amylose_*n*_	time in [s]	GOSTSHYP (%)	cavity (%)	1st step (%)	2nd step (%)
1	23	43.9	6.5	26.6	10.8
2	86	48.5	3.1	31.8	13.6
4	229	58.9	2.5	38.2	18.3
8	1264	66.7	1.0	42.8	22.9
16	4801	70.9	0.8	44.2	25.9
32	18845	71.9	1.1	44.2	26.6
48	44960	70.1	1.7	43.2	25.1
64	83825	68.1	2.6	41.3	24.2

a Fraction of the overall computation time spent on GOSTSHYP is shown, including the individual fractions of the cavity construction and both GOSTSHYP steps in the integral-direct algorithm.

The overall trends are similar to the calculations in which RI-*J* was used. The total computation times are drastically reduced for larger systems, as to be expected. Here, GOSTSHYP can make up about 70% of the computation time, even though this percentage decreases again for larger systems. The pressure simulation on the electronic structure of the amylose_64_ system takes less than a day on a single CPU, which is quite an astonishing feature. Only here, in combination with the highly efficient MARI-*J* approximation, does GOSTSHYP become the most time-consuming step in the calculation.

**Figure 4:**
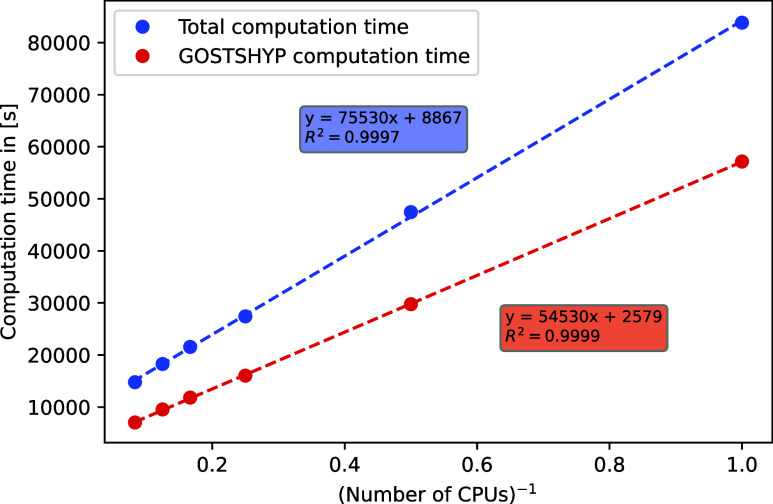
Computation time for amylose_64_ plotted against the inverse number of CPUs. All calculations were performed on the PBE/def2-SV(P) level of theory with MARI-*J*. Depicted here are the total computation time (blue) as well as the computation time for the GOSTSHYP part (red).

We close this section by examining the speed up achieved by our parallel implementation. Both integral-direct steps (blue and pink parts highlighted in Figure 1) have been parallelized with open multiprocessing (OpenMP) and can thus be run on multiple CPUs. In order to assess the quality of our parallel implementation of GOSTSHYP, we have run the calculations on amylose_64_ on the PBE/def2-SV(P) level of theory with MARI-*J* on up to 12 CPUs and benchmarked the timings. The results are shown in Figure 4, where we have plotted the computation time against the inverse number of CPUs. This plot has the advantage that both the efficiency increase and the computational overhead are clearly visible.

The almost perfect description through a linear regression (*R*^2^ = 0.9997 for the entire calculation and *R*^2^ = 0.9999 for the GOSTSHYP part) indicates that the parallelization does indeed accelerate the calculation as expected. There is a relatively large overhead of almost 2.5 h (8867 s) for the entire calculation, which cannot be further reduced by an increase in CPUs. This includes the set up of the GOSTSHYP cavity and MARI-*J*, as well as some steps within the SCF procedure. If we examine only the fraction of the computation time that GOSTSHYP requires, we see a more favorable behavior. The computational overhead of GOSTSHYP is only about 40 min (2579 s), with most of this time at about 35 min (2166 s) being required for the construction of the FINE cavity. The stark increase of computational time required for this step for larger systems was already indicated in Tables 2 and 3.

To conclude, the integral-direct GOSTSHYP algorithm presented in this work was shown to be highly efficient and parallel. The effects of high pressure on the electronic structure of a large molecule such as amylose_64_ with more than 1000 atoms and well over 10,000 basis functions can now be calculated in a few hours using relatively inexpensive hardware.

### 4.2 Pressure-Dependent Binding Energy of C_60_ Fullerene and a Corannulene Pincer System

**Figure 5:**
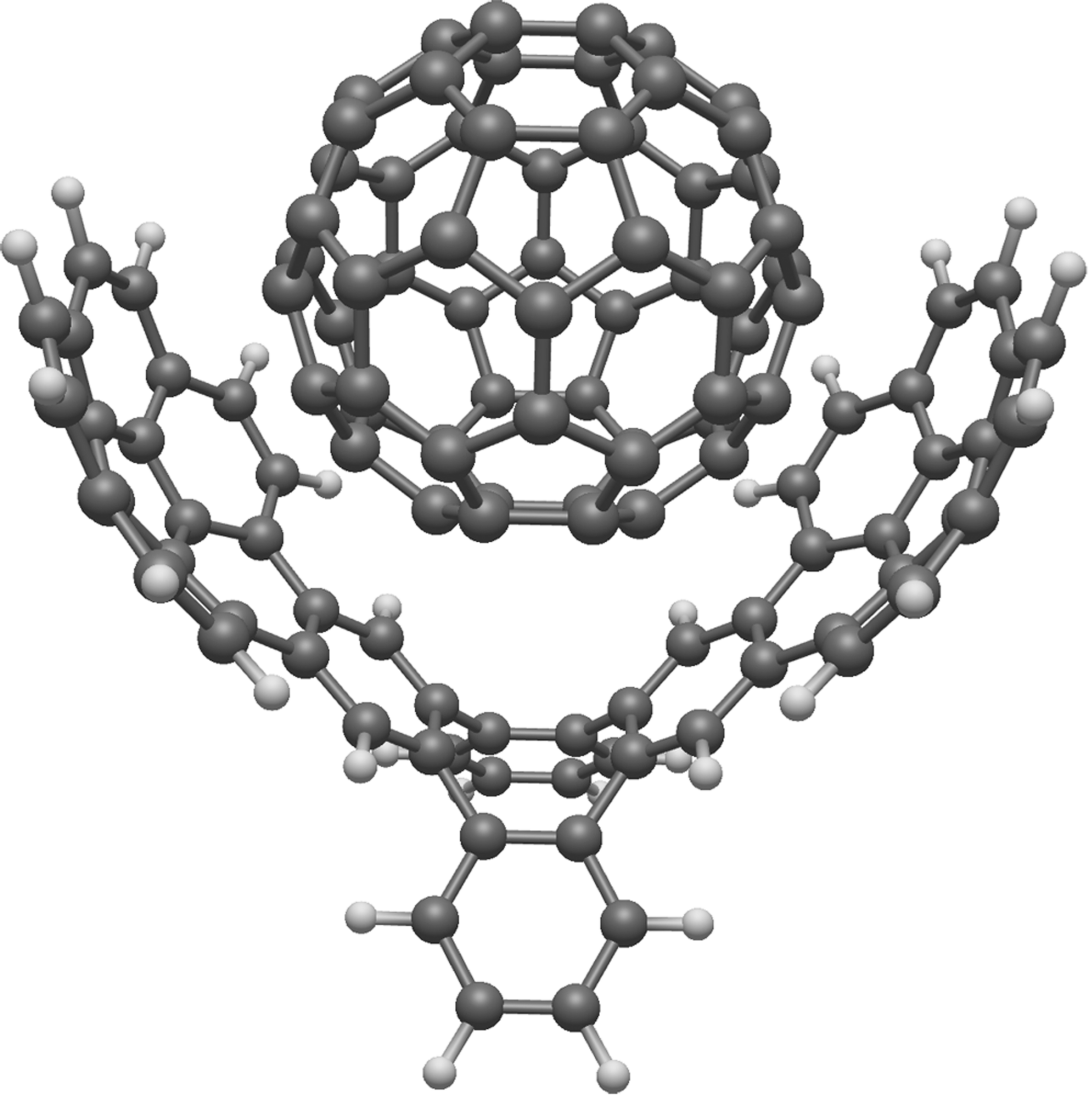
Molecular structure of the supersystem consisting of a Buckminster fullerene (C_60_) and a corrannulene pincer system.

In Section 2.6, the advantages and disadvantages of a variety of different cavity constructions for GOSTSHYP were discussed. In this section, we evaluate the influence of the choice of cavity on the results of a GOSTSHYP calculation. For this purpose, we examine how external pressure affects the binding energy *E*_B_ of a system consisting of a Buckmister fullerene (C_60_) and a corannulene pincer system. The molecular structure of the supersystem is depicted in Figure 5. Several computational studies have been carried out to investigate the binding energy in the absence of high pressure effects of this system over the past years.^87−93^

We start this investigation by comparing our results with those found in the literature for the system in a vacuum. At the B3LYP-D3/def2-SVP level of theory, we find that the binding energy is 147.2 kJ mol^–1^. This is in relatively good agreement with calculations performed by Grimme on the TPSS-D3/def2-QZVP’ level of theory, which yielded 152.8 kJ mol^–1^.^91^ Furthermore, the difference between our binding energy and one empirically calculated by Sure and Grimme of 118.7 ± 2.5 kJ mol^–1^ is only about 25%.^89^

**Figure 6:**
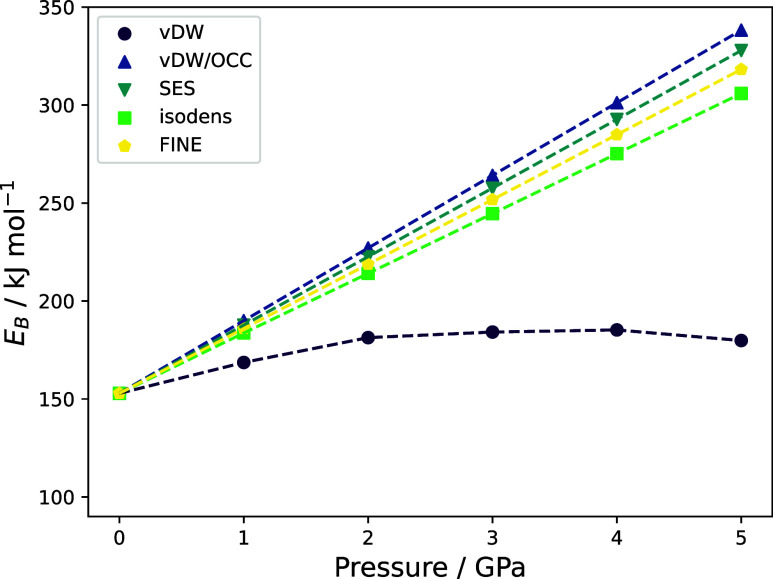
Pressure dependence of the binding energy *E*_B_ for the C_60_@pincer system for the different types of cavities discussed in Section 2.6.

Having confirmed that we obtain a reasonable result in the absence of high pressure effects, we now examine the pressure dependence of the relaxed binding energy. We use GOSTSHYP in combination with the five different types of cavities presented in Section 2.6 and restrict our investigation here to pressures of up to 5 GPa. Using the geometries we obtained from the pressure-free calculations, we computed single-point energies on the B3LYP-D3/def2-SVP level with GOSTSHYP. The BSSE was accounted for with counterpoise corrections. The resulting binding energies are plotted against the external pressure in Figure 6.

For all types of cavities investigated here except for the vdW cavity, we find a linear dependency with a similar slope between 30 and 40 kJ mol^–1^ GPa^–1^. These results are also presented in Table 4, giving additional information about the SCF convergence. Therein, we show the average number of SCF cycles *n̅*_SCF_ as well as the number of negative GOSTSHYP amplitudes *n̅*_*p*_*j*_ < 0_ detected at the end of the calculation for all systems. Please note that for some systems, a limited number of negative amplitudes do occasionally occur during the iterative procedure. As long as they eventually vanish, they are not listed here.

**Table 4:** Pressure Dependence of the Relaxed Binding Energy Calculated on the B3LYP-D3/def2-SVP Level of Theory with GOSTSHYP for the Different Cavities Discussed in Section 2.6^a^

	C_60_		pincer		C_60_@pincer		
cavity	*n̅*_SCF_	*n̅_*p*_*j*__*_<0_	*n̅*_SCF_	*n̅*_*p*_*j*_<0_	*n̅*_SCF_	*n̅*_*p*_*j*_<0_	∂*E*_B_/∂*p* in [kJ mol^–1^ GPa^–1^]
isodensity^67^	12	0	6	0	17	0	30.6
FINE^54^	12	0	14	0	17	0	33.0
SES^62^	6	0	7	0	9	0	33.0
vDW^51^	6	0	18	37	151	260	
vDW/OCC	6	0	7	0	9	0	37.1

a For each molecule, the average number of SCF cycles *n̅*_SCF_ and the average number of detected negative amplitudes *n̅*_*p_j_*<0_ are presented. For the vDW cavity, *E*_B_ did not exhibit a linear pressure dependence, therefore we omitted the slope ∂*E*_B_/∂*p* given in units of [kJ mol^–1^ GPa^–1^].

Clearly, the vdW cavity is an outlier concerning both the SCF convergence and the pressure dependence of the binding energy. It is the only type of cavity investigated here that exhibits negative amplitudes, both for the isolated pincer system and the C_60_@pincer supersystem. As described in previous literature,^47^ the negative amplitudes lead to an erratic convergence of the SCF procedure, which can be seen in the highly increased number of SCF iterations. Furthermore, the binding energy depicted in Figure 6 does not increase linearly with the external pressure. All of this indicates that the vdW cavity is ill-equipped to handle these type of systems due to the presence of undesirable crevices. Please note that the OCC appears to solve these issues entirely if an appropriate extension radius is chosen.

Given the erratic nature of the vdW cavity for these systems, how can we ensure that no cavity-related artifacts appear in calculations with GOSTSHYP? Luckily, the method itself appears to have an in-built diagnostic tool, with negative amplitudes indicating an unsuitable cavity. Please note that their presence is not limited to vdW cavities. For all types of cavities, negative amplitudes may emerge if certain parameters such as the solvation radius or the electron density cutoff^94,95^ are not properly set. The occurrence of negative amplitudes should always be interpreted as a sign that cavity-related artifacts might be present.

**Figure 7:**
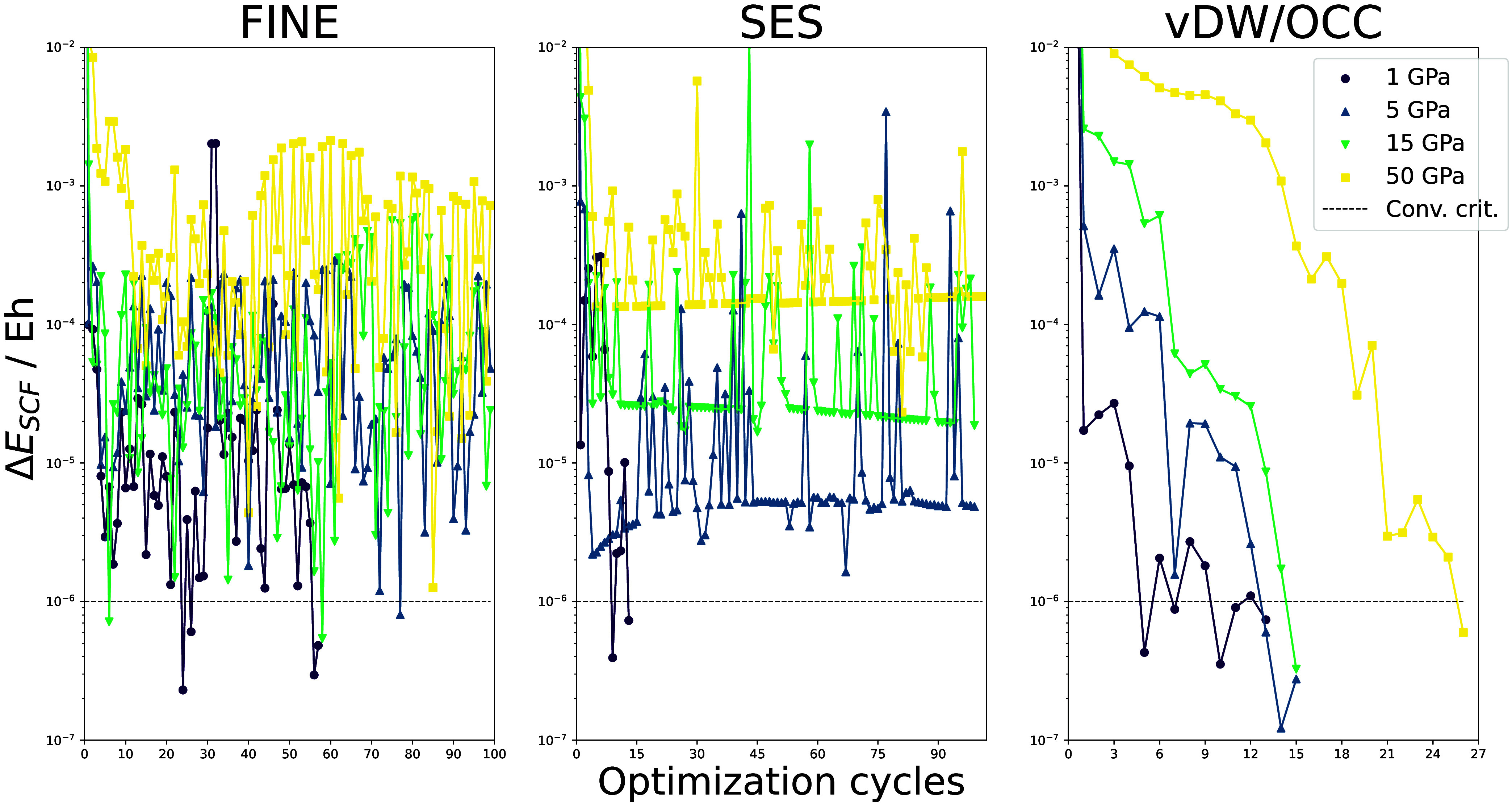
Convergence behavior for GOSTSHYP geometry optimizations using the vdW/OCC, FINE, and SES cavities. Depicted here is the change in the electronic energy during each geometry optimization step. All calculations were carried out on the B3LYP-D3/def2-SVP level of theory.

Having investigated the cavity-related convergence issues for single-point calculations, we conclude this section by examining the influence on the geometry optimization. We limit this discussion to three types of cavities: vdW/OCC, the FINE cavity, and the SES. Out of these three cavity types, only vdW/OCC has fully consistent gradients in our implementation. Derivatives of the FINE cavity are implemented, but the related PES is not smooth (see the discussion in Section 2.6.5 for more information). For the SES, cavity derivatives are not available in our implementation and they are thus approximated to be zero. For the C_60_@pincer supersystem, the convergence of the geometry optimizations is presented in Figure 7.

Using the FINE cavity leads to significant convergence problems due to the discontinuous nature of the related PES. Even for calculations with a small external pressure, the geometry optimizations generally do not converge. A similar picture presents itself for the SES. Here, the geometry optimizations converged for calculations with small external pressure, but they became erratic for higher pressures. Only the vdW/OCC cavity leads to a rapid and comparatively smooth convergence for all external pressures investigated here.

For the investigation on the pressure-dependence of the binding energy of C_60_ and the corannulene pincer system, we thus now focus our concluding remarks on the results from calculations performed with the vdW/OCC cavity. Here, the pressure-dependence obtained from the single-point calculations was 37.07 kJ mol^–1^ GPa^–1^. The geometry optimization only has minimal effect, increasing this value by about 1.5% to a total of 37.65 kJ mol^–1^ GPa^–1^. Still, as expected, the supramolecular interaction between C_60_ and the pincer is considerably more stable in high pressure conditions.

Having assessed the cavity-related effects on both single-point calculations and geometry optimizations, it becomes very clear that in our current implementation, vdW/OCC is the cavity of choice. It yields similar results to those obtained from the other cavities for single-point calculations and has a fully consistent gradient, making reliable geometry optimizations possible.

## 5 Conclusions

In recent years, GOSTSHYP has emerged as a reliable method for the investigation of high pressure effects on molecular and electronic structures. Compared to other approaches, it has a variety of desirable features, including its relatively straightforward integration into the toolbox of quantum chemical methods. The striking similarities to the widely established continuum solvation model COSMO further indicate that it can be seamlessly implemented into most modern quantum chemistry software. However, two problems have plagued previous GOSTSHYP implementations: unfavorable memory demand with increasing system size and the emergence of ill-defined amplitudes for the GOSTSHYP model potentials. In the context of this work, we have introduced a novel GOSTSHYP implementation which tackles both of these issues.

The new algorithm presented herein is integral-direct and only stores small intermediate quantities in memory. In combination with a novel approximate integral-screening technique, the effects of high pressure on extended molecular structures may now be assessed with minimal computational resources. We have implemented this new algorithm into the Turbomole program suite, where it will be available in the upcoming official release version 7.9. To demonstrate the efficiency of our new GOSTSHYP implementation, we have performed benchmark calculations on increasingly large chains of α-d-glucose units. We showed that using our new implementation, molecules with more than 1000 atoms and well over 10,000 basis functions can now be calculated in only a few hours using a relatively inexpensive hardware setup.

Finally, we have investigated the pressure dependence of the binding energy between a C_60_ Buckminster fullerene and a corannulene pincer system. This computational study had not been possible with previous GOSTSHYP implementations due to artifacts introduced by the ill-defined amplitudes. Using our novel implementation, we could assess that the source of these artifacts are unphysical regions in the cavity. By analyzing different types of cavities and carefully examining their advantages and disadvantages, we have constructed a new variant that yields very reliable results for such a type of computational study. This van der Waals surface with an outer cavity correction (vdW/OCC) is the only type of cavity that has a fully consistent geometry gradient, but does not introduce any ill-defined amplitudes in our implementation. We believe that this vdW/OCC cavity can also facilitate future investigations of high pressure effects on molecular and electronic structure.

## Figures and Tables

**Figure 8:**
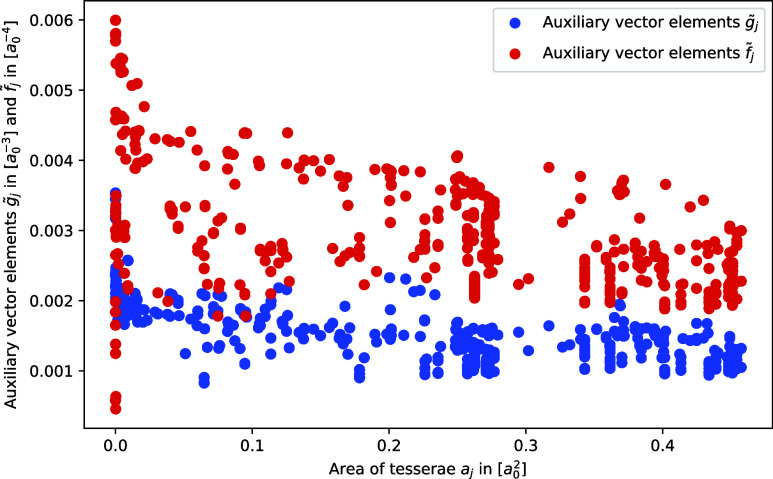
Auxiliary vector elements *g̃*_*j*_ and *f̃*_*j*_ plotted against the area of their respective tesserae after a converged SCF calculation on anthracene at the CAM-B3LYP/def2-SVP level of theory. Both *g̃*_*j*_ and *f̃*_*j*_ are narrowly distributed without any significant outliers. This is a prerequisite for any approximate screening technique employed for GOSTSHYP.

## Appendix

### A: Integral Evaluation

#### A.1: Three-Center Overlap Integrals

In this section, we want to address how to efficiently evaluate all relevant integrals for GOSTSHYP. Since they are three-center overlap integrals, we briefly reiterate how to tackle two-center overlap integrals over Cartesian GTOs. A normalized Cartesian GTO is defined as

33

Here, *N*_μ_ is the normalization constant and ζ_μ_ is the exponent. The GTO is centered at **R**^μ^ and **a**^μ^ is a vector which contains integers corresponding to the type of Cartesian function |μ⟩ is representing, with |**a**^μ^| = 0 being an *s*-type function, |**a**^μ^| = 1 a *p*-type function and so forth. Please note that this notation refers to Cartesian functions and that spherical harmonics can be constructed as linear combinations thereof.^96^ The two-center overlap integral over two normalized *s*-type GTOs may then be computed as

35

where we have used the Gaussian product theorem to reduce the complexity of the problem.^97^ Instead of an integration over two Gaussian functions centered at **R**^μ^ and **R**^ν^, respectively, we only require one integration over a Gaussian centered at **P**. Additionally, we need to define the total exponent ζ and the reduced exponent σ of the Gaussian charge distribution

36

37

38

For a three-center overlap integral, we can use the Gaussian product theorem again to reduce the problem to a single integration of a Gaussian function

40

The area-dependent GOSTSHYP exponents ω_*j*_ were previously defined in eq 4, the total exponent of this charge distribution is ζ_*j*_, the reduced exponent is σ_*j*_, and the center of charge is denoted as **P**_*j*_

41

42

43

The remaining integral in eq 40 may be evaluated as

44

Please note that the three-center overlap integrals over three *s*-type functions are the starting point for all integrals that are relevant for GOSTSHYP. Recurrence relations for integrals over functions associated with higher angular momentum quantum numbers may be calculated using the Gauss–Hermite polynomial approach or, alternatively, the Obara–Saika recursion relations.^98,99^ In this work, we have implemented the former approach.

#### A.2: Efficient Approximate Screening

In recent work,^46,47^ the normalization constant *N*_*j*_ was neglected as it cancels out for any segment in the evaluation of the GOSTSHYP energy in eq 5. As mentioned before, we used it in this work nevertheless, as it facilitates the screening of both the *g*_μν,*j*_ and *f*_μν,*j*_ integrals. How does the normalization affect the evaluation of three-center integrals? Depending on the chosen cavity parametrization, some tesserae might be associated with very small areas, leading to extremely small auxiliary vector elements *g̃*_*j*_ and *f̃*_*j*_. Such elements can still have a significant contribution to the GOSTSHYP energy because the amplitude *p*_*j*_ depends on the inverse of *f̃*_*j*_. Therefore, small elements of *g*_μν,*j*_ and *f*_μν,*j*_ cannot generally be neglected if no normalization is used. In ref (47), a very thorough, but rather involved screening technique was introduced to circumvent this problem. However, we find that the use of normalized Gaussians in the integral evaluation is sufficient to solve this issue and adapt the common approach to approximate integral screening for extended molecular structures by neglecting integrals below a certain threshold in our implementation.^82,96,100^

A prerequisite for an efficient approximate screening of GOSTSHYP three-center integrals is that the integral is both *bounded* and *separable* with respect to a subset of its components.^96^ In order to introduce our approximate screening approach, we first start comparing (eqs 35 and 40). We note that

45

and

46

from which we can deduce that the following inequality must hold

47

The three-center integral is bounded according to eq 47 and may be brought into a separable form if we consider the maximum element of the vector containing the normalization constants *N*_max_ = max(*N*_*j*_)

48

As such, we have found a bounded and separable form which may be used to screen the three-center overlap integrals using the two-center overlap integrals over *s*-type functions, similar to established methods for four-center integrals.^82,96,100^

However, these conditions alone are not sufficient to ensure an efficient approximate screening for GOSTSHYP. The three-center integrals are employed in (eqs 7 and 8) to construct the auxiliary vectors **g̃** and **f̃**. In order to calculate the GOSTSHYP energy contribution, their individual elements are contracted as

49

according to eq 5. This shows that an individual contribution to the electronic energy becomes very large if *g̃*_*j*_ ≫ *f̃*_*j*_. Furthermore, it implies that a contribution may be significant, even if *g̃*_*j*_ is very small (namely, if the respective element *f̃*_*j*_ is also very small). For an efficient approximate screening technique, it is therefore very beneficial if the values contained in the auxiliary vectors **g̃** and **f̃** are all of a similar order of magnitude. This can be ensured by the use of the normalization constants *N*_*j*_ in the integral evaluation of eq 40 and all subsequent steps. To demonstrate this, we consider the two limits of very large and very small area segments.

For a very large area segment *a*_*j*_, we find that ω_*j*_ becomes small according to eq 4. This corresponds to an estimate of ω_*j*_ ≪ ζ in eq 40, which simplifies the equation to

50

This is clearly well-screened by the bound established in eq 48, as *N*_max_ ≥ *N*_*j*_ e^–^ω_*j*_ (**r**_*j*_ – **P**)^2^.

Perhaps more important is the limit of very small area segments. In vdW cavities, these segments are often found in the undesirable crevices and they can also be associated with severe convergence problems. As such, approximate screening techniques should not introduce large errors for these segments in particular. The limit ω_*j*_ ≫ ζ simplifies eq 40 to

51

This expression is not dependent on ω_*j*_ anymore, which implies that even for arbitrarily small area segments, the limit does not diverge. Please note the similarities between eqs 50 and 51, particularly considering that ω_*j*_ is small in the former, while ζ is small in the latter.

#### A.3: Analysis for Auxiliary Vectors

We conclude this work by presenting an analysis of the elements contained in the auxiliary vectors of an extended molecular system. As discussed in Appendix A.2, the use of the normalization constant *N*_*j*_ in integrals should lead to elements in **g̃** and **f̃** that are of similar order of magnitude. This is a prerequisite for the efficient approximate screening technique presented in this work, as previously discussed. While the limits presented in eqs 50 and 51 suggested that this is the case, we will demonstrate this on an example.

As a system, we chose the anthracene molecule and converged an SCF calculation on the CAM-B3LYP/def2-SVP level of theory with a medium-sized DFT grid (grid 3).^73,75,79,80,101^ For GOSTSHYP, we used a vdW cavity parametrized via Lebedev grids (grid 6),^53^ with solvation radii of 1.3 Å for hydrogen and 2.0 Å for carbon.

The distribution of values in **g̃** and **f̃** as a function of the area in the respective tessera are depicted in Figure 8. Median, maximum and minimum values for the tesserae areas, as well as the elements of **g̃** and **f̃** are presented in Table 5. While the tesserae areas can become very small, the elements of both auxiliary vectors are very narrowly distributed. Furthermore, there is no clear trend between increasing tessera area and an in- or decrease in the respective elements of the auxiliary vectors. This indicates that through the use of the normalization constant, indeed, the elements of **g̃** and **f̃** may be screened using the criterion established in eq 48.

**Table 5:** Analysis of Tesserae Areas, as well as Elements of the Auxiliary Vectors **g̃** and **f̃**^a^

	area in [*a*_0_^2^]	**g̃** in [*a*_0_^–3^]	**f̃** in [*a*_0_^–4^]
median	0.2615	0.00146	0.00287
maximum	0.4574	0.00354	0.00600
minimum	5.09 × 10^–11^	0.00083	0.00046

a Evaluated for anthracene at the CAM-B3LYP/def2-SVP level of theory. While the area of segments can become very small, the values of auxiliary vectors are very narrowly distributed around the median.

## Supporting Information Available

Molecular coordinates (xyz format) for all molecules discussed in Section 4.2 (ZIP)
